# Development of a Web-App for the Ecological Momentary Assessment of Dietary Habits among College Students: The HEALTHY-UNICT Project

**DOI:** 10.3390/nu14020330

**Published:** 2022-01-13

**Authors:** Martina Barchitta, Andrea Maugeri, Giuliana Favara, Roberta Magnano San Lio, Paolo Marco Riela, Luca Guarnera, Sebastiano Battiato, Antonella Agodi

**Affiliations:** 1 Department of Medical and Surgical Sciences and Advanced Technologies “GF Ingrassia”, University of Catania, 95123 Catania, Italy; andrea.maugeri@unict.it (A.M.); giuliana.favara@gmail.com (G.F.); robimagnano@gmail.com (R.M.S.L.); agodia@unict.it (A.A.); 2 Department of Mathematics and Informatics, University of Catania, 95123 Catania, Italy; paolo.riela@unict.it (P.M.R.); luca.guarnera@phd.unict.it (L.G.); battiato@dmi.unict.it (S.B.)

**Keywords:** ecological momentary assessment, college students, COVID-19 pandemic, healthy behaviors

## Abstract

The transition from adolescence to adulthood is a critical period for the development of healthy behaviors. Yet, it is often characterized by unhealthy food choices. Considering the current pandemic scenario, it is also essential to assess the effects of coronavirus disease-19 (COVID-19) on lifestyles and diet, especially among young people. However, the assessment of dietary habits and their determinants is a complex issue that requires innovative approaches and tools, such as those based on the ecological momentary assessment (EMA). Here, we describe the first phases of the “HEALTHY-UNICT” project, which aimed to develop and validate a web-app for the EMA of dietary data among students from the University of Catania, Italy. The pilot study included 138 students (mean age 24 years, SD = 4.2; 75.4% women), who used the web-app for a week before filling out a food frequency questionnaire with validation purposes. Dietary data obtained through the two tools showed moderate correlations, with the lowest value for butter and margarine and the highest for pizza (Spearman’s correlation coefficients of 0.202 and 0.699, respectively). According to the cross-classification analysis, the percentage of students classified into the same quartile ranged from 36.9% for vegetable oil to 58.1% for pizza. In line with these findings, the weighted-kappa values ranged from 0.15 for vegetable oil to 0.67 for pizza, and most food categories showed values above 0.4. This web-app showed good usability among students, assessed through a 19-item usability scale. Moreover, the web-app also had the potential to evaluate the effect of the COVID-19 pandemic on students’ behaviors and emotions, showing a moderate impact on sedentary activities, level of stress, and depression. These findings, although interesting, might be confirmed by the next phases of the HEALTHY-UNICT project, which aims to characterize lifestyles, dietary habits, and their relationship with anthropometric measures and emotions in a larger sample of students.

## 1. Introduction

During the transition from adolescence to adulthood, the development of healthy behaviors represents the foundations for ensuring health and wellbeing across a lifetime. Yet, this period is often characterized by a decline in physical activity levels, a worsening of overall diet quality, and a high risk of gaining weight [1]. Unhealthy behaviors may interact with each other, further exacerbating their negative effect on human health. For instance, many college students make unhealthy food choices, which may make them more vulnerable to long-term adverse health outcomes. Moreover, it has been proposed how other unhealthy behaviors—such as a sedentary lifestyle—might affect diet quality by acting on the social context of eating occasions [2]. The complexity of this issue requires the development of innovative tools for simultaneously assessing behaviors in real time and in the real world. In fact, traditional dietary assessment methods have specific limitations that should be considered. The use of dietary records, for instance, can yield high-quality data, but requires a high level of motivation from respondents [3,4]. By contrast, the 24 h dietary recall imposes relatively little burden on respondents, but all information is based on their memory and the skills of their interviewers. Despite being relatively simple, cost-effective, and time-efficient, even food frequency questionnaires (FFQs) are not exempt from inaccuracies [5]. To overcome some of these limitations, the ecological momentary assessment (EMA) represents an innovative approach to evaluate emotions and behaviors at the exact time they occur in real life. Repeated sampling strategies involve both event- or signal-contingent EMA, which can be useful to minimize recall bias and to investigate how microprocesses might influence real-world behaviors.

With this purpose, different EMA tools have the potential to assess dietary habits and other behaviors in epidemiological studies and to outperform traditional methods [6]. Potential areas of application of EMA range from the study of dietary patterns among healthy people to the management of patients with eating or metabolic disorders. The recent technological innovation, moreover, gave the opportunity to develop many web-based tools that may increase the study size, improve the quality of data, and reduce the costs. For instance, the study by Berkman and colleagues demonstrated that an electronic EMA could improve the response rate to assess the consumption of energy-dense foods compared with a paper-and-pencil tool [7].

The adolescence and the subsequent period are crucial for developing healthy lifestyles and for making responsible choices about foods and beverages. This period is also important for the development of so-called emotional eating, which is characterized by eating more food and/or consuming more calories in response to negative emotions rather than to a feeling of hunger [8]. On the other hand, it has been reported how dietary habits are important for academic outcomes and students’ performance [9]. Indeed, the adherence to a healthy dietary pattern—such as Mediterranean Diet—may lead to better academic performance, as well as improve quality of life and mental health status [10]. Given that, previous studies showed promising findings about the application of EMA to assess dietary habits among students [1,11,12]. Despite these interesting prospects, however, many studies applied EMA only to characterize snacking, food craving, and overeating episodes among students [13,14,15]. Only a few, instead, applied EMA with a more general view of evaluating the main determinants of food choices and dietary habits of students [16,17]. In fact, a proper understanding of the reasons behind food choices could be helpful to guide effective interventions to improve the quality of diet throughout life [18,19,20]. In our opinion, an approach like the EMA produces a large amount of data about concurrent events and exposures that are fundamental for investigating factors associated with dietary habits. This type of data could also be important to evaluate the impact of the coronavirus disease-19 (COVID-19) pandemic on healthy behaviors [6]. In fact, both lockdown and social distancing have led to important changes in the lifestyles of people. Students, in particular, are affected by the COVID-19 pandemic through the spread of several unhealthy behaviors, such as sedentary activities, poor dietary habits, and reduced sleep quality, which in turn affect their stress levels and depression [21,22]. During the current pandemic, restrictions and lockdown procedures contributed to inadequate food choices, resulting in a poorer quality of diet for students [23].

In this scenario, we designed the “Healthy diet and lifestyles among University of Catania Students: a mobile Ecological Momentary Assessment approach for health promotion, HEALTHY-UNICT” project to characterize lifestyles, dietary habits, and their relationship with anthropometric measures and emotions in a sample of students from the University of Catania (Catania, Italy). In the present article, the primary objective was to describe the development of a web-app for the EMA of dietary habits among students, and the subsequent pilot study was designed to evaluate the validity and usability of this tool. Next, we also reported findings from the pilot study regarding a survey of the impact of the COVID-19 pandemic on student’s lifestyles and their psychological status.

## 2. Materials and Methods

### 2.1. Study Design

**Figure 1:**
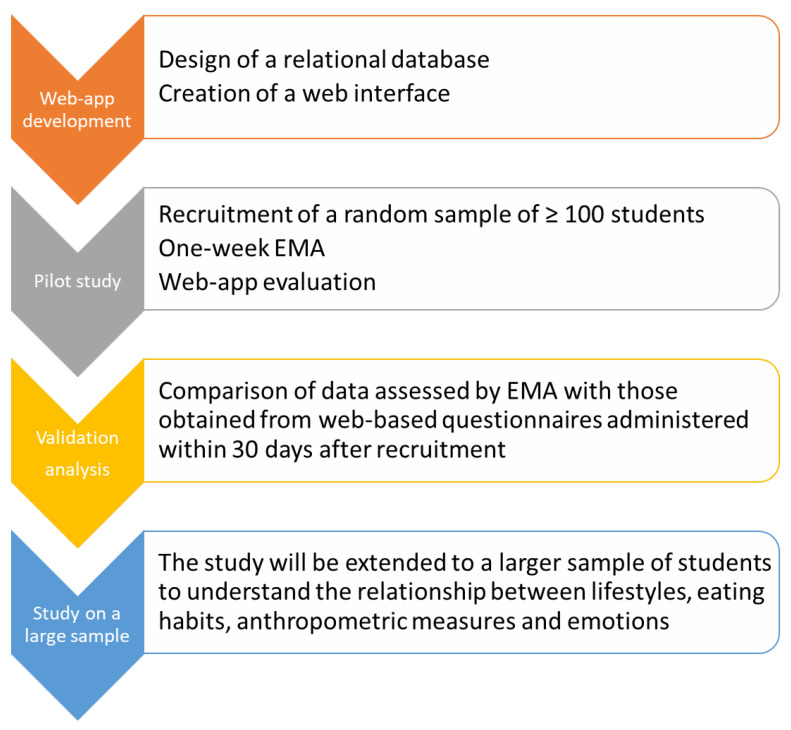
Summary of all phases of the HEALTHY-UNICT project.

All the phases of the HEALTHY-UNICT project are summarized in Figure 1. We first developed a web-app for the EMA of dietary habits, other behaviors, and emotions among students from the University of Catania (Catania, Italy). Next, we conducted a pilot study on a convenience sample of students to evaluate the validity and usability of the web-app for the EMA of dietary habits. To achieve this aim, the pilot study was conducted during the second semester of the academic year 2020–2021 (i.e., from April to June 2021). The study protocol was approved by the ethics committee (CE Catania 1, n.70/2021/PO del Registro dei pareri del CE, prot. N. 16857 dell’08.04.2021) and performed according to the Declaration of Helsinki. All students attending Hygiene and Public Health courses of the Bachelor’s or Master’s degrees in Biological Sciences, Medicine, and Health professions were invited to participate in the pilot study. Particularly, students were invited to take part in the study through verbal explanation of the project, on how to use the web-app, on how they will receive notifications, on how to respond to questionnaires, and on the amount of time required to respond. Later, an email was sent to each of them. After being informed about the purpose and procedures of the study, students who agreed provided their written informed consent to participate in the study. At recruitment, participants were asked to fill out a web-based questionnaire about socio-demographic factors, anthropometric measures, and behaviors. Next, participants were asked to use the web-app for a week prior to fill out a web-based food frequency questionnaire (FFQ) within 30 days after recruitment. The validity of the web-app was tested against the FFQ, in terms of correlations and weighted Kappa agreement.

Regarding sample size, at least 125 students were needed to obtain an expected kappa of 0.6 with a minimum acceptable kappa of 0.3, a significance level of 0.05, a statistical power of 0.8, and a drop-out rate of 10%.

At the end of the pilot study, students were also asked to compile two surveys regarding the usability of the web-app and the impact of COVID-19, respectively.

### 2.2. Development of the Web-App

**Figure 2:**
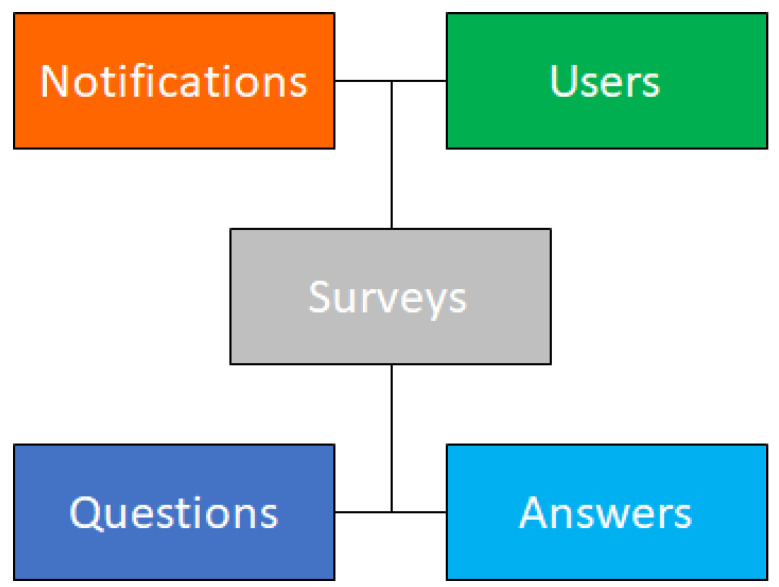
Generic scheme of the database structure.

The development started with the design of a relational database including tables for user’s data, groups of questionnaires, questions, and closed answers. The database consisted of several tables, including Notifications, Users, Surveys, Questions, and Answers. Figure 2 shows a generic scheme of the database structure. Front-end and back-end were defined through a set of different PHP scripts. The back-end interface was designed to allow researchers to define questionnaires with questions and answers and to schedule individual questionnaires over time. The data were stored in a well-designed relational database management system (RDBMS). MySQL was chosen to be managed by PHP scripts and for determining the relationships between users and scheduled questionnaires. The Notifications table showed the progress of every user in the completion of questionnaires and the Survey table defined the question/answer scheme in a scheduled program. Finally, the Users table showed the details of anonymized participants after their registration. In order to respect the database rules, we added other tables able to join N-N relationships. The front-end was defined by a responsive web interface that guides the user step by step from registration to account confirmation up to the completion of the surveys. To ensure full understanding of questions and surveys, the front-end was created in Italian language. Once the registration phase with the user data was completed, an activation link was sent via email. When an account was activated, the app created a personalized questionnaire program for the new user. The user interface showed the available questionnaires based on the survey schedule. A notification via email was sent to the users at different times of the day if they had questionnaires to fill out. The notification included a personal link containing a hash that allowed users to directly access their personal home without entering login credentials.

### 2.3. Design of the Ecological Momentary Assessment

**Figure 3:**
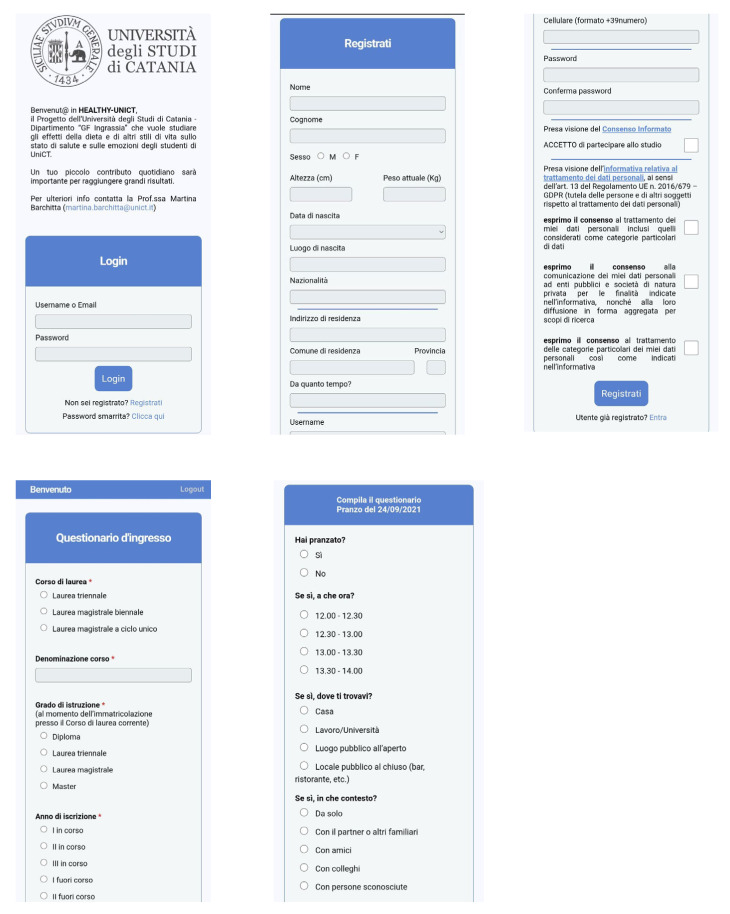
Participant views of the web-app from a smartphone. The web-app was conceived in Italian language to ensure full understanding of questions and surveys.

Next, each participant was asked by email to complete a seven-day EMA of behaviors, based on a signal-contingent approach at semifixed intervals (i.e., 07:00–10:00, 10:00–13:00, 13:00–16:00, 16:00–19:00, 19:00–22:00). To cover the main daily activities while minimizing the burden for participants, students received by mail five prompts per day and each data entry required about 2–3 min. As such, a potential for 35 data entries for each participant was possible with the EMA. The EMA scheme is summarized in the Supplementary Table S1, while some screenshots of the web-app are reported in Figure 3. Regarding dietary habits, participants were prompted to indicate foods and beverages consumed during the day (i.e., at breakfast, morning and afternoon snack, lunch, and dinner) from a list of 38 food groups based on nutrient profiles and culinary use. For each food group, the frequency of consumption was weighted for the number of eating occasions; as such, the value ranged from 0 (food/beverage not consumed at all) to 1 (food/beverage consumed at every meal). Participants were also asked to report the physical and social contexts in which they had their meals. The remaining prompts asked participants to report on water consumption, sleep duration and quality, physical activity, smoking habits, and other daily activities.

### 2.4. Food Frequency Questionnaire

To test the validity of the EMA tool, a web-based 95-item semiquantitative FFQ [9,24] was administered within 30 days after recruitment. The FFQ consisted of 95 items grouped into the same 38 food groups used for the EMA. For each item, students were asked to indicate the frequency of consumption and the portion size, as described elsewhere [9,24]. For each food group, the intake was calculated as the sum of the products between the frequency of consumption and portion size of related food items. To allow the comparison with data obtained from the web-app, these values were scaled to a 0–1 range.

### 2.5. Evaluation of Usability of the Web-App

At the end of the pilot study, a survey was launched to evaluate the feasibility and usability of the web-app. The survey was designed ad hoc, also considering questions used by previous studies [11,25], to collect information needed for further improving the web-app in the next phases of the project. The survey was based on 19 statements, which covered domains related to objectives and information on the study, usability of the web-app, number and impact of prompts, and commitment required. For each item, participants were asked to indicate their degree of agreement, based on a five-point Likert scale from “completely agree” to “completely disagree”. The items were analyzed separately and by computing an aggregated scale, with higher values reflecting optimal feasibility and usability.

### 2.6. Assessment of the Impact of the COVID-19 Pandemic

The current COVID-19 pandemic raised the interest in evaluating its impact on dietary habits and other behaviors. For this reason, in the framework of the pilot study of the HEALTHY-UNICT project, we decided to launch a survey of changes in behaviors and psychological conditions of students after a year of the pandemic. The survey was designed ad hoc on the basis of previous questionnaires [22,26,27]. This survey was based on 21 items, which covered domains related to sleep, physical activities, walking, sedentary behaviors, smoking cigarettes, consumption of foods and beverages, weight, stress, and depression. For each item, participants were asked to indicate the impact of the pandemic, based on a five-point Likert scale from “extremely negative impact” to “extremely positive impact”. The items were analyzed separately and by computing an aggregate scale, with higher values reflecting a negative impact of the pandemic.

### 2.7. Statistical Analysis

Prior to analysis, the normal distribution of all variables was checked using the Kolmogorov-Smirnov test. Descriptive statistics were used to characterize participants, using frequency and percentage or mean and standard deviation. Next, three methods were used to validate the web-app against the web-based FFQ for the assessment of dietary data. We first evaluated the correlations between measures obtained from the two different tools by calculating Spearman’s rank correlation coefficients. The quartile cross-classification and the weighted Cohen’s kappa were further applied to evaluate the ability of the web-app to adequately rank students according to their dietary data. For the cross-classification analysis, more than 50% of participants should be classified in the same quartile, while those classified into the opposite quartile should not exceed 10% [28]. For the weighted kappa analysis, the value should be above 0.4 [28]. For the two scales related to the web-app usability and to the impact of COVID-19 pandemic, we computed the Cronbach’s alpha as a measure of internal consistency and checked the dimensionality through exploratory factor analysis (EFA). After verifying the assumptions of the EFA (Kaiser-Meyer-Olkin measure and Barlett test of sphericity), principal component analysis (PCA) with varimax rotation was used. Principal components were extracted and interpreted according to the Scree plot inspection and factor loadings >0.32. A *p* < 0.05 was considered statistically significant and statistical analyses were mainly performed using SPSS software (version 22.0, SPSS, Chicago, IL, USA).

## 3. Results

### 3.1. Study Population

The pilot study was conducted on 138 students enrolled in Bachelor’s degree (52.9%) or Master’s degree (47.1%) courses at the University of Catania. All students completed the recruitment questionnaire, and their characteristics are summarized in Table 1. Overall, 92.8% of students answered to all 35 prompts received during the EMA, while only 4.4% completed less than 30 data entries. Moreover, 95.7% of students completed the FFQ administered within 30 days after recruitment; these data together with those obtained through the EMA were used to validate the ability of the web-app for assessing dietary habits.

### 3.2. Validity of the Web-App

**Figure 4:**
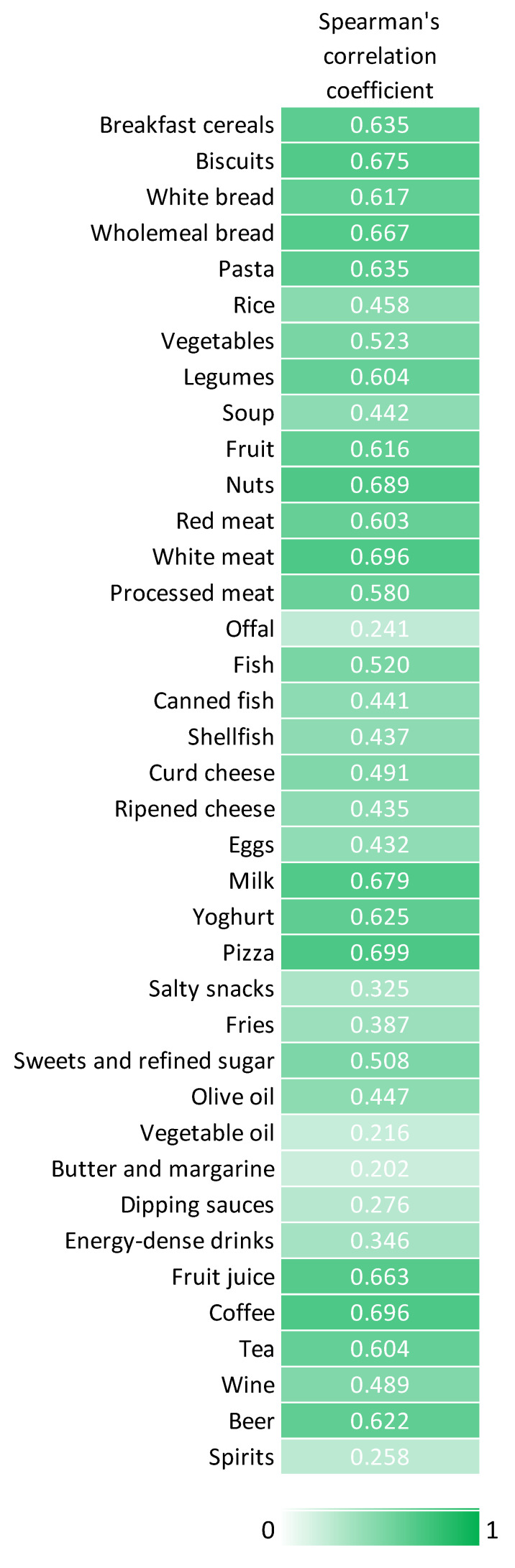
Spearman’s correlation coefficients of dietary data obtained from the web-app and the food frequency questionnaire.

**Figure 5:**
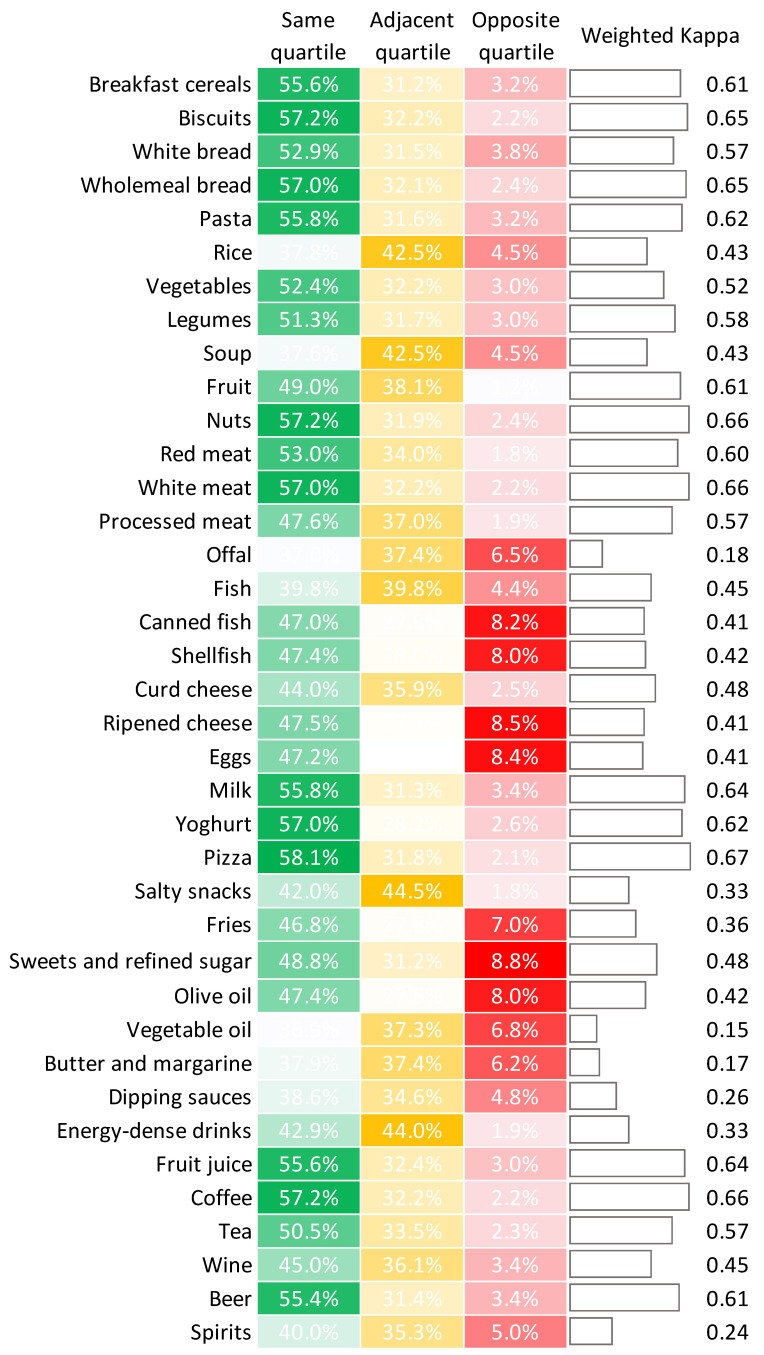
Cross-classification and weighted-kappa analyses of dietary data obtained from the web-app and the food frequency questionnaire. For the cross-classification analysis, more than 50% of participants should be classified into the same quartile, while those classified into the opposite quartile should not exceed 10%. For the weighted kappa analysis, the value should be above 0.4.

A total of 132 students had sufficient data to be included in the validation phase, while 6 students did not fill out the FFQ. No significant differences between included and excluded students were evident. In general, there was moderate correlation between data obtained from the web-app and the FFQ. Figure 4 displays the Spearman’s correlation coefficients for each food category. These values ranged from 0.202 for butter and margarine to 0.699 for pizza. Figure 5 summarizes the results from the cross-classification and weighted-kappa analyses. The percentage of students classified into the same quartile ranged from 36.9% for vegetable oil to 58.1% for pizza. Accordingly, these percentages were above 50% for 17 food categories. By contrast, the percentage of students classified in the opposite quartile ranged from 1.2% for fruits to 8.8% for sweets and refined sugar. In line with these findings, the weighted-kappa values ranged from 0.15 for vegetable oil to 0.67 for pizza, and most of the food categories showed values above 0.4. Weighted-kappa values below 0.4 were observed for fries, salty snacks, energy-dense drinks, dipping sauces, spirits, offal, butter and margarine, and vegetable oil.

### 3.3. Usability of the Web-App

For the 19-item scale related to the web-app usability, the Cronbach α was 0.725 and a three-component solution explained 62.3% of the total variance. The first component was loaded by items related to the usability of the web-app and to the number of prompts. The second component was mainly loaded by items related to the burden for participants. The third component was mainly loaded by items related to the interest in the research and to information received before the study. Table 2 reports the level of agreement of participants with each statement and the mean score assessed on a five-point Likert scale. Accordingly, the feasibility and usability seemed good, with the highest scores reported for participants’ interest in the research and for the completeness of the information received. In line with these results, the overall satisfaction of students was high, as highlighted by an average score of 4.4 (SD = 0.2).

### 3.4. The Impact of the COVID-19 Pandemic

For the 21-item scale related to the impact of the COVID-19 pandemic, the Cronbach α was 0.681 and a three-component solution explained 63.2% of the total variance. The first component was loaded by items related to physical activity, sleep quality, and consumption of unhealthy foods. The second component was loaded by items related to sleep duration and to the consumption of healthy foods. The third component was mainly loaded by items related to sedentary activities, stress, and depression. Overall, the pandemic seems to have had a moderate impact on students, as indicated by an average score of 3.2 (SD = 0.3) on a five-point Likert scale. The domains most affected included sedentary activities, level of stress, and depression, with everyone having a score equal to or higher than 4 (Table 3). Accordingly, the percentage of students reporting a negative impact was 84.8% for sedentary at work, 82.6% for sedentary during the weekend, 79.0% for the level of stress, and 73.9% for depression. By contrast, no significant impact was evident for the remaining domains.

## 4. Discussion

In this article, we describe the first phases of the HEALTHY-UNICT project, which aims to provide a better understanding of the food choices that college students make and insights for individual- and population-level prevention interventions.

The study started with the development of a tool to conduct the EMA of students’ behaviors with a particular focus on dietary habits. As described for the first time in 2008 by Shiffman et al. [29], this kind of assessment allows the collection of data about behaviors and experiences in the natural environment, in real time, and over a certain period [29]. For these reasons, the EMA of dietary habits could be applied in the field of nutritional epidemiology to achieve different goals [6]. The tool that we present here was developed according to our previous systematic review of EMA methods applied to the dietary assessment in epidemiological studies [6]. This review highlighted that the research in this field began in 2013 with the independent work of two research groups [11,12]. Taken together, these studies confirmed that EMA tools give the opportunity to determine complex behaviors and their relationships with social and intrapersonal factors. In line with this evidence, other research groups applied the EMA to understand the main determinants associated with snacking [13], food craving [7,30], and over-eating episodes [14]. Yet, despite the increasing application of EMA to assess specific dietary factors and/or events, few studies applied it to understand the complex relationships between different behaviors (i.e., diet, physical activity, sedentary, smoking status, and so on), social factors, and psychosocial conditions.

In line with most of the previous studies, our EMA tool consisted of a web-app, working on several devices (e.g., smartphones, tablets, and personal computers), to collect information about dietary habits, sleeping, physical activities, and other daily activities. Our EMA was based on a signal-contingent approach, which notified participants to record their activities at semi-fixed intervals. Although this approach did not assess activities at the exact time they occured, it allowed to record information within a recent interval of time. It is worth mentioning that the signal-contingent approach was very common in this kind of study [1,13,15,16,17,31,32,33]. The contraposed approach, defined as event-contingent, required a greater engagement by participants, who had to remember to record information at each eating occasion [12,34]. In our pilot study, the EMA lasted a week, a period consistent with what was observed in our previous review [6]. To improve the assessment of dietary data, some studies consisted of more than one wave of EMA [1,16,17,35,36,37,38]. Among them, the Social impact of Physical Activity and nutRition in College (SPARC) study was one of a kind [16]. The authors developed a mobile EMA app to collect data about the consumption of specific foods and drinks from a list of sixteen items. The main aims of the SPARC study were to assess eating behaviors and other daily activities of about 1500 students over an academic year [1], and to investigate how friendship networks and emotions affect eating habits, physical activity, and weight [17].

Our web-app was designed to collect dietary data on 38 food categories. In the pilot study, this tool was validated against a traditional FFQ. It is worth noting that a few studies compared their EMA tools with traditional assessment methods [1,39]. Moreover, differences between studies discouraged the comparison with our study. For instance, Chmurzynska et al. evaluated the validity of a signal-contingent EMA tool for assessing the consumption of high-fat foods [39]. The application of this EMA tool improved the quality of dietary data if compared with those obtained through a traditional retrospective questionnaire [39]. Similarly, researchers of the SPARC study evaluated their EMA app against 24 h recall [1]. The authors demonstrated that total dietary intake assessed through the app reflected eating choices captured by the 24 h recall, but match rates varied in relation to specific food categories [1]. The pilot study discussed here shows that this web-app could really allow the EMA of students’ dietary habits in large size and prospective studies. Indeed, dietary data obtained through the web-app exhibited significant correlations with those collected through the FFQ. Moreover, apart from a few food categories, the web-app correctly classified students according to dietary data. The exceptions were related to foods and beverages that were not commonly consumed (i.e., fries, salty snacks, energy-dense drinks, dipping sauces, spirits, offal, butter and margarine, and vegetable oil). A possible solution to improve the assessment of these food categories is to extend the EMA period or, alternatively, to conduct more than one wave.

When interpreting our findings, other limitations should be considered. A potential selection bias cannot be completely excluded because participants were invited from a source population of college students in health science and medicine courses. Accordingly, the study population consisted of participants with a high educational level, and it was mostly made of women. For these reasons, it can be assumed that all participants have at least a moderate interest in nutrition. Moreover, the web-app was developed to assess dietary habits in general and not to estimate the intake of specific nutrients. This choice was made to reduce the burden for participants. Similarly, the web-app was designed to assess foods consumed at semi-fixed intervals coinciding with meals. Thus, dietary data did not represent the total dietary intake, but only that related to main meals and snacks. However, the web-app represents a valid tool to capture the distribution of food choices among students and to investigate the main determinants associated with healthy or unhealthy dietary habits. Beyond its validity, the web-app exhibited good feasibility and usability. In fact, the overall satisfaction of students was high, especially for their interest in the research and for the adequacy of information received before the study. Domains related to the web-app features and to the burden for participants also received good scores. Some students, however, suggested to add more categories to the list of foods. In our opinion, further improvements are still possible, such as the use of SMS instead of email, and the project’s dissemination via social network, press releases, and leaflets. On the other hand, however, the web-app has the potential to create and launch short surveys to pre-recruited people. This might help to address new and sudden needs for public health professionals. For instance, in the pilot study, we launched a brief survey on the impact of the COVID-19 pandemic on students’ behaviors. Although the limited sample size precluded us from drawing definitive conclusions, the main domains affected by the pandemic seemed to be those related to sedentary behaviour, stress, and depression. These preliminary findings, even if reflecting what was achieved in other settings [40,41,42,43], need to be confirmed and further investigated.

This can be done in the next phase of the HEALTHY-UNICT project, which plans to recruit a larger sample of students from different degree courses of the University of Catania. The general opinion is that students’ food choices are moving towards a Western and unhealthy diet [10]. Moreover, it has been established that students who experienced negative emotions tended to consume more high-calorie and junk foods. On the contrary, those who felt positive tended to consume more fruits and vegetables [17]. Despite this evidence, however, little is still known about the relationships between emotions and food choices, especially among college students. This represents the main goal of the HEALTHY-UNICT project, which seeks to characterize lifestyles and dietary habits among students and to investigate their relationship with anthropometric measures and emotions.

## 5. Conclusions

In conclusion, we developed a web-app based on the EMA of dietary habits among students, which represented a promising tool for the collection of data in real time and in the real world. This tool showed good validity and usability and its application will be extended to a larger sample to uncover the main determinants of food choices of young adults. In the coming future, the achievement of this objective could provide new insights for designing prevention strategies and interventions at the individual and population levels.

## Figures and Tables

**Table 1:** Characteristics of the study population.

Characteristics (*n* = 138)	Mean (SD)	Frequency (%)
Age, years	24.0 (4.2)	
Gender		
Male		34 (24.6%)
Female		104 (75.4%)
Type of degree course		
Bachelor’s degree		73 (52.9%)
Master’s degree		65 (47.2%)
Type of student		
Resident		33 (23.9%)
Commuter		37 (26.8%)
Non-resident		68 (49.3%)
Smoking status		
Smoker		21 (5.1%)
Ex-smoker		7 (5.1%)
Non-smoker		110 (79.7%)
BMI, kg/m^2^	22.9 (5.0)	
BMI categories		
Underweight		19 (13.9%)
Normal weight		86 (62.8%)
Overweight		25 (18.2%)
Obese		7 (5.1%)

**Table 2:** Level of agreement with statements related to the research and to the usability of the web-app.

Questions	Completely Disagree	Disagree	Uncertain	Agree	Completely Agree	Score ^a^
1. The aim of the study was interesting and stimulating	-	-	-	32.9%	67.1%	4.7 (0.5)
2. The information received was clear	-	1.4%	1.4%	26.0%	71.2%	4.7 (0.5)
3. The information received allowed to immediately use the web-app	1.4%	-	4.1%	39.7%	54.8%	4.5 (0.7)
4. The web-app was easy to use	-	-	4.1%	30.1%	65.8%	4.6 (0.6)
5. The web-app was funny	-	2.7%	16.4%	38.4%	42.5%	4.2 (0.8)
6. The web-app was boring	46.6%	32.9%	15.1%	5.5%	-	4.2 (0.8)
7. The number of prompts was adequate	2.7%	4.1%	9.6%	50.7%	32.9%	4.1 (0.9)
8. The number of prompts was excessive	42.5%	39.7%	12.3%	5.5%	-	4.2 (0.9)
9. I answered to all prompts for seven days	-	1.4%	6.8%	23.3%	68.5%	4.6 (0.7)
10. In general, I answered to all daily prompts	2.7%	4.1%	2.7%	28.8%	61.6%	4.4 (0.9)
11. The web-app was clear and simple	-	1.4%	2.7%	37.0%	58.9%	4.5 (0.6)
12. It was simple to answer from the smartphone	-	-	5.5%	28.8%	65.8%	4.6 (0.6)
13. I used my personal computer to answer	75.3%	13.7%	5.5%	2.7%	2.7%	4.6 (0.9)
14. The answers required a lot of time	42.5%	35.6%	15.1%	6.8%	-	4.1 (0.9)
15. The answers stopped my daily activities	41.1%	38.4%	11.0%	6.8%	2.7%	4.1 (1.0)
16. The study was too long	41.1%	41.1%	13.7%	4.1%	-	4.2 (0.8)
17. The study required a lot of commitments	61.6%	30.1%	6.8%	1.4%	-	4.5 (0.7)
18. The commitment required was adequate	2.7%	-	6.8%	54.8%	35.6%	4.2 (0.8)
19. I would recommend it	-	-	2.7%	39.7%	57.5%	4.5 (0.6)

^a^ For questions with a negative sense (e.g., question 6), the score was computed by giving increasing values from completely agree (1) to completely disagree (5).

**Table 3:** The impact of the COVID-19 pandemic on behaviors.

Questions	1 (Positive)	2	3 (No Impact)	4	5 (Negative)	Score
1. Hours of sleep per day	2.9%	29.0%	42.8%	21.7%	3.6%	2.9 (0.9)
2. Ease to fall asleep	12.3%	19.6%	46.4%	20.3%	1.4%	2.8 (1.0)
3. Number of days with vigorous activities for at least ten minutes	7.2%	18.1%	30.4%	20.3%	23.9%	3.4 (1.2)
4. Number of days with moderate activities for at least ten minutes	9.4%	31.2%	24.6%	21.7%	13.0%	3.0 (1.2)
5. Number of days with walking for at least ten minutes	10.1%	11.6%	21.0%	31.9%	25.4%	3.5 (1.3)
7. Total time spent sitting for at least 10 min in a working day	2.2%	2.2%	10.9%	29.0%	55.8%	4.3 (0.9)
8. Total time spent sitting for at least 10 min in the week-end	0.7%	2.2%	14.5%	36.2%	46.4%	4.3 (0.8)
9. If smoker, number of cigarettes per day	9.5%	9.5%	33.3%	33.3%	14.3%	3.3 (1.2)
10. Consumption of fruits	1.4%	26.1%	67.4%	5.1%	-	2.8 (0.6)
11. Consumption of vegetables	6.5%	26.8%	65.2%	1.4%	-	2.6 (0.6)
12. Consumption of legumes	3.6%	20.3%	74.6%	1.4%	-	2.7 (0.5)
13. Consumption of cereals	2.2%	16.7%	76.8%	3.6%	0.7%	2.8 (0.5)
14. Consumption of fats	0.7%	8.0%	51.4%	36.2%	3.6%	3.3 (0.7)
15. Consumption of fish	1.4%	16.7%	73.9%	5.1%	2.9%	2.9 (0.6)
16. Consumption of dairy products	0.7%	4.3%	68.8%	25.4%	0.7%	3.2 (0.6)
17. Consumption of meat	3.6%	8.0%	72.5%	15.2%	0.7%	3.0 (0.6)
18. Drinking alcohol	26.8%	15.2%	48.6%	9.4%	-	2.4 (1.0)
19. Body weight	2.9%	20.3%	31.9%	35.5%	9.4%	3.3 (1.0)
20. Depression	-	2.2%	23.9%	51.4%	22.5%	4.0 (0.7)
21. Level of stress	0.7%	-	20.3%	53.6%	25.4%	4.0 (0.7)

## Data Availability

The data presented in this study are available on request from the corresponding author.

## Supplementary Materials

The following is available online at https://www.mdpi.com/article/10.3390/nu14020330/s1, Table S1: The EMA scheme indicating questions, answers, and the number of prompts per week.
