# Supplementary material for: Development of a Web-App for the Ecological Momentary Assessment of Dietary Habits among College Students: The HEALTHY-UNICT Project
Source: Nutrients. 2022 Jan 13;14(2):330. doi: 10.3390/nu14020330 (PMC8779738; doi:10.3390/nu14020330)
Supplement: Supplementary file 1 [file nutrients-14-00330-s001.zip › nutrients-1545979-supplementary.pdf]

Supplementary Table S1. The EMA scheme indicating questions, answers and the number of prompts per week

Questions	Answers	Prompts per week
**Did you have breakfast?**	**Yes**<br>**No**	5
What time?	Time	
Where?	Home<br>Work/University<br>Public open space<br>Public closed space	
What was the social context?	Alone<br>With partner or family members<br>With friends<br>With colleagues<br>With unknown people	
Could you indicate foods and beverages consumed?	Multiple choice from a food list	
**Did you have lunch?**	**Yes**<br>**No**	5
What time?	Time	
Where?	Home<br>Work/University<br>Public open space<br>Public closed space	
What was the social context?	Alone<br>With partner or family members<br>With friends<br>With colleagues<br>With unknown people	
Could you indicate foods and beverages consumed?	Multiple choice from a food list	
**Did you have dinner?**	**Yes**<br>**No**	5
What time?	Time	
Where?	Home<br>Work/University<br>Public open space<br>Public closed space	
What was the social context?	Alone<br>With partner or family members<br>With friends<br>With colleagues<br>With unknown people	
Could you indicate foods and beverages consumed?	Multiple choice from a food list	
**Did you have morning snack?**	**Yes**<br>**No**	3
What time?	Time	
Where?	Home	

	Work/University<br>Public open space<br>Public closed space	
What was the social context?	Alone<br>With partner or family members<br>With friends<br>With colleagues<br>With unknown people	
Could you indicate foods and beverages consumed?	Multiple choice from a food list	
**Did you have afternoon snack?**	**Yes**<br>**No**	3
What time?	Time	
Where?	Home<br>Work/University<br>Public open space<br>Public closed space	
What was the social context?	Alone<br>With partner or family members<br>With friends<br>With colleagues<br>With unknown people	
Could you indicate foods and beverages consumed?	Multiple choice from a food list	
**What are you doing?**	**Working or studying**<br>**Watching tv, listening music, reading**<br>**Eating or drinking**<br>**Doing physical activities**<br>**Other**	3
Where?	Home<br>Work/University<br>Public open space<br>Public closed space	
What is the social context?	Alone<br>With partner or family members<br>With friends<br>With colleagues<br>With unknown people	
Are you sitting down?	Yes<br>No, I’m standing<br>No, I’m walking<br>No, I’m running	
**How many liters of water did you drink yesterday?**	**<1**<br>**1**<br>**1.5**<br>**2**<br>**>2**	3
Other drinks?	Yes<br>No	

If yes, which ones?	Wine<br>Beer<br>Liquor<br>Spirits<br>Sweetened drink<br>Coffee<br>Milk<br>Tea<br>Fruit juice<br>Other	
**What time did you fall asleep yesterday?**	**Time**	
What time did you get up?	Time	
How many times did you wake up during the night?	0<br>1<br>2<br>3<br>4<br>5-8<br>9+	3
How did you sleep?	Much worse than usual<br>Worse than usual<br>As usual<br>Better than usual<br>Much better than usual	
**Did you engage in vigorous physical activity yesterday?**	**Yes**<br>**No**	
For how many minutes?	Minutes	
Did you engage in moderate physical activity yesterday?	Yes<br>No	3
For how many minutes?	Minutes	
For how many minutes did you walk yesterday?	Numero	
How fast?	Slowly<br>Moderately<br>Quickly	
For how many minutes did you sit yesterday?	Minutes	
**Did you smoke yesterday?**	**Yes**<br>**No**	
If yes, what did you smoke?	Cigarettes<br>Electronic cigarette<br>Heat-not-burn cigarettes<br>Other	2
How many cigarettes?	1-5	
	5-10<br>11-15<br>16-20<br>+20	
